# An Antedrug of the CXCL12 Neutraligand Blocks Experimental Allergic Asthma without Systemic Effect in Mice*

**DOI:** 10.1074/jbc.M112.449348

**Published:** 2013-02-28

**Authors:** François Daubeuf, Muriel Hachet-Haas, Patrick Gizzi, Vincent Gasparik, Dominique Bonnet, Valérie Utard, Marcel Hibert, Nelly Frossard, Jean-Luc Galzi

**Affiliations:** From the ‡ Laboratoire d'Innovation Thérapeutique, UMR 7200 CNRS/Université de Strasbourg, Faculté de Pharmacie, 74 Route du Rhin, 67401 Illkirch, France,; the ¶ Laboratoire de Biotechnologie et Signalisation Cellulaire, Ecole de Biotechnologie de Strasbourg, UMR 7242 CNRS/Université de Strasbourg, Ecole Supérieure de Biotechnologie, Bd. Sébastien Brant, 67412 Illkirch, France, and; the § Laboratory of Excellence MEDALIS, Université de Strasbourg, 67400 Illkirch, France

**Keywords:** Inflammation, Chemokines, Soft Drugs

## Abstract

The chemokine receptor CXCR4 and its chemokine CXCL12 are involved in normal tissue patterning but also in tumor cell growth and survival as well as in the recruitment of immune and inflammatory cells, as successfully demonstrated using agents that block either CXCL12 or CXCR4. In order to achieve selectivity in drug action on the CXCR4/CXCL12 pair, in particular in the airways, drugs should be delivered as selectively as possible in the treated tissue and should not diffuse in the systemic circulation, where it may reach undesired organs. To this end, we used a previously unexploited Knoevenagel reaction to create a short lived drug, or soft drug, based on the CXCL12-neutralizing small molecule, chalcone 4, which blocks binding of CXCL12 to CXCR4. We show that the compound, carbonitrile-chalcone 4, blocks the recruitment of eosinophils to the airways in ovalbumin-sensitized and challenged mice *in vivo* when administered directly to the airways by the intranasal route, but not when administered systemically by the intraperitoneal route. We show that the lack of effect at a distant site is due to the rapid degradation of the molecule to inactive fragments. This approach allows selective action of the CXCL12 neutraligands although the target protein is widely distributed in the organism.

## Teaser

**Background:** The chemokine CXCL12 and its receptor CXCR4 are widely distributed and contribute to the physiopathology of inflammation.

**Results:** Recruitment of eosinophils in the inflamed airway is selectively attenuated by short lived antagonists that block CXCL12-mediated activation of CXCR4.

**Conclusion:** CXCL12/CXCR4 signaling regulates local leukocyte-mediated inflammation.

**Significance:** Antedrugs of neutraligands allow dissecting the physiological role of chemokines, especially when expression occurs in multiple tissues.

## Introduction

Chemokines are small proteins that play critical roles in the development and function of various tissues in vertebrates. In the adult, they regulate the directional migration of leukocytes under normal and pathological conditions. As a rather general rule, chemokines and their G protein-coupled receptors display redundancy and binding promiscuity (*i.e.* several chemokines may bind to the same receptor set) (1), whereas a few chemokines play a pivotal and non-redundant homeostatic role. A singular case is that of the CXCL12/SDF1 chemokine and its receptor CXCR4, which are both conserved during evolution from jawless fish to humans and appear essential during normal embryogenesis and organogenesis (2–4). CXCL12 is constitutively expressed by stromal, epithelial, and endothelial cells in primary lymphoid organs (including bone marrow and thymus) and secondary lymphoid organs, such as spleen and ganglia (5). Disruption of either the *CXCL12* (5) or the *CXCR4* (4) gene is lethal during mouse embryogenesis, illustrating the prominent role of CXCL12 and CXCR4 in the patterning of embryonic tissue formation through progenitor cell migrations. Suppression of CXCL12/X4 interaction upon treatment with granulocyte(-macrophage) colony-stimulating factor (GM-CSF or G-CSF) (6, 7) or with the selective CXCR4 antagonist AMD 3100 promotes neutrophilia (8). In the adult, CXCR4 and CXCL12 maintain stem cell niches in the bone marrow and contribute to the proliferation of hematopoietic progenitors (9, 10).

CXCL12 and CXCR4 are also important players in pathophysiological situations (11–14), including AIDS (15–17), the unusual form of neutropenia reported as WHIM syndrome (18–20), or carcinogenesis (11, 14, 21). In addition, CXCR4 and CXCL12 are also implicated in inflammation. They contribute to promoting transendothelial migration of lymphocytes (22) and invasion of inflamed tissues, as illustrated in the airways of animal models of asthma (23–27), in the pulmonary vasculature in pulmonary arterial hypertension (28), and in fibroproliferative tissue in a murine model of obliterative bronchiolitis after heterotopic tracheal transplantation (29).

CXCL12 and CXCR4 were long thought to be the exclusive interactors of each other until the recent discovery that the orphan G protein-coupled receptor, CXCR7, also binds CXCL12 as well as CXCL11 (30, 31). CXCR7 is expressed by endothelial cells and cardiomyocytes and is essential in heart development (32, 33). CXCR7 does not elicit clear responses to CXCL12 but clearly associates with the CXCR4 protein to modulate its sensitivity for CXCL12 (33, 34).

The physiological and pathophysiological importance of CXCL12, CXCR4, and CXCR7 has prompted the launching of drug discovery programs aiming at blocking HIV entry, inhibiting cancer cell proliferation, or reducing inflammatory responses. The most advanced compound is the CXCR4 antagonist AMD 3100, which has been approved for treatment of lymphoproliferative disorders (Plerixafor®). It displays efficacy in humans in mobilizing CXCR4^+^ progenitor cells (10, 35–38) upon acute administration. Use of AMD 3100 is currently being evaluated for other therapeutic indications, such as glioblastoma and the WHIM syndrome (39, 40). It is, however, endowed with side effects, mainly cardiotoxicity (41), which is an expected problem if one considers the multiplicity of tissues expressing CXCR4 as well as the variety of diseases in which CXCR4 is implicated. According to a recent report (42), AMD 3100 is presumed to act as an agonist of the CXCR7 receptor, a property that might account for potential secondary effects of AMD 3100.

**SCHEME 1.**
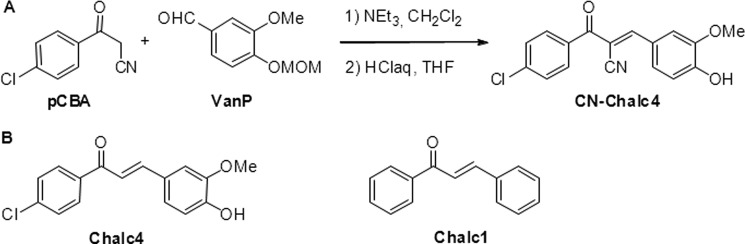
*A*, synthesis of CN-chalcone 4 (*CN-Chalc4*) following a Knoevenagel condensation of pCBA with 3-methoxy-4-(methoxymethyloxy)benzaldehyde (*VanP*). *B*, structure of reference compounds chalcone 4 (*Chalc 4*) and unsubstituted chalcone (*Chalc 1*).

An alternative strategy consists in preventing the agonist-receptor interaction by neutralizing the endogenous ligands. In this context, we have identified a compound that belongs to this category of pharmacological agents (*i.e.* a small neutralizing compound binding to CXCL12), chalcone 4 (Scheme 1), which prevents CXCL12 binding either to CXCR4 or CXCR7 (21, 23, 24, 26, 43, 44). Chalcone 4 blocks responses of CXCR4 to CXCL12 *in vitro* without affecting the basal level receptor activity and displays anti-inflammatory effects in a murine model of asthma *in vivo*.

In order to favor desired local anti-inflammatory action of the neutraligand at the expense of undesired distant effects, we explored the possibility of generating a short lived neutraligand prone to efficient biodegradation before it distributes in the body and reaches unwanted tissues. To this end, we describe here the synthesis of a molecule using previously unexploited Knoevenagel/retro-Knoevenagel reactions to generate so called soft drugs or antedrugs (45). Characterization of the functional properties of the new short lived carbonitrile-chalcone, *in vitro* and *in vivo*, shows that it is active when administered locally and inactive after systemic administration.

## EXPERIMENTAL PROCEDURES

### 

#### 

##### Chemistry

Reagents were obtained from commercial sources and used without any further purification. Thin-layer chromatography was performed on silica gel 60F_254_ plates. Flash chromatography was performed on silica gel (puriFlash® 30 μm, Interchim) or C18 (puriFlash® 30 μm, Interchim) prepacked columns on a SpotII Ultima from Armen. NMR spectra were recorded on a Bruker AV400 spectrometer. Chemical shifts (δ) are reported in ppm, and coupling constants (*J*) are expressed in Hz. Analytical HPLC analyses were performed on an Eclipse XBD-C18 column (5 μm, 46 × 150 mm; Agilent) using the following conditions: flow rate, 1 ml/min; Solvent A, 0.1% aqueous TFA; Solvent B, 0.1% TFA in CH_3_CN; gradient, 5–100% B developed over 15 min; detection at 220/254/365 nm. Retention times (*t_R_*) from analytical reverse phase HPLC are reported in min. LC/MS spectra were obtained on an Agilent HPLC single quadrupole spectrometer (1200RRLC/1956b-SL) equipped with a THERMO Hypersyl column (1.9 μm, 1 × 30 mm) using an Agilent Multimode ion source. High resolution mass spectrometry spectra were obtained on an Accurate-Mass Q-ToF spectrometer from Agilent using electrospray ionization. For (*E*)-2-(4-chlorobenzoyl)-3-(4-hydroxy-3-methoxyphenyl)acrylonitrile (CN-chalcone 4)^6^, *p*-chloro-benzoyl-acetonitrile (1 g, 5.606 mmol) and 3-methoxy-4-(methoxymethyloxy) benzaldehyde (1.1 g, 5.606 mmol) were dissolved in dry CH_2_Cl_2_ (15 ml). Dry NEt_3_ (78 μl, 0.56 mmol) and pulverized activated 4-Å molecular sieves (1 g) were added. The mixture was stirred at room temperature, monitoring the progress of the reaction by TLC. After 20 h, the molecular sieve was filtered off, and the organic layer was concentrated *in vacuo* to dryness. The residual crude orange solid was recrystallized from aqueous EtOH to afford (*E*)-2-(4-chlorobenzoyl)-3-(3-methoxy-4-(methoxymethoxy)phenyl) acrylonitrile as a yellow solid (1.01 g, 50% yield). *R_f_* = 0.38 (heptane-ethyl acetate: 7–3); mp = 140–1 °C; ^1^H NMR (CDCl_3_): *d* 3.51 (s, 3H), 3.96 (s, 3H), 5.32 (s, 2H), 7.23 (d, *J* = 8.6 Hz, 1H,), 7.43 (dd, *J* = 8.6, 2.1 Hz, 1H), 7.48 (d, *J* = 8.5 Hz, 2H), 7.83 (d, *J* = 8.5 Hz, 2H), 7.88 (d, *J* = 2.1 Hz, 1H), 8.00 (s, 1H); ^13^C NMR (CDCl_3_): *d* 55.9, 56.3, 95.2, 106.5, 112.8, 116.2, 117.9, 125.7, 128.3, 129.1, 130.8, 134.8, 139.8, 150.3, 151.8, 156.1, 188.1.

(*E*)-2-(4-Chlorobenzoyl)-3-(3-methoxy-4-(methoxymethoxy)phenyl)acrylonitrile was dissolved in THF (10 ml) in the presence of a 1 n HCl aqueous solution (5 eq, 14 ml). The resulting mixture was stirred at room temperature, monitoring the progress of the conversion by HPLC. After 12 h, the solvent was removed under reduced pressure, and the residue was dissolved in CH_2_Cl_2_ and washed with water until neutralization. The solvent was removed, and deprotected CN-chalcone 4 was recovered as a yellow solid in quantitative yield (886 mg). *R_f_* = 0.27 (heptane-ethyl acetate: 8–2); mp = 162–3 °C (recrystallized from aqueous EtOH); ^1^H NMR (CDCl_3_): δ 3.98 (s, 3H), 6.26 (br s, 1H), 7.01 (d, *J* = 8.3 Hz, 1H,), 7.41 (dd, *J* = 8.3, 2.1 Hz, 1H), 7.48 (d, *J* = 8.6 Hz, 2H), 7.82 (d, *J* = 8.6 Hz, 2H), 7.91 (d, *J* = 2.1 Hz, 1H), 8.00 (s, 1H); ^13^C NMR (CDCl_3_): *d* 56.4, 105.9, 111.5, 115.3, 118.1, 124.7, 129.1, 129.5, 130.7, 134.6, 139.6, 147.0, 151.4, 156.0, 188.0. reverse phase HPLC purity >97%; high resolution mass spectrometry calcd. for C_17_H_13_ClNO_3_ 314.0506; found: 314.0518 (M+ H)^+^. Chalcone 1 and chalcone 4 were provided by the French national chemical library.

##### In Vitro and in Vivo Experiments

Chalcone stock solutions were prepared in sterile DMSO and then stored at −20 °C until use. The human chemokines CXCL12 and CXCL12-TR were purchased from Alamc. The cAMP biosensor CAMYEL was kindly provided by Dr. Lily Jiang (Dallas, TX).

##### Determination of Solubility and Stability

Solubility and stability studies were performed on a Gilson HPLC system with a photodiode array detector, an autosampler, and a Valco injector. Data acquisition and processing were performed with Trilution LC version 2.0 software. Measurements were carried out at 21 ± 1 °C. A 2.6-μm Kinetex column (50 × 4.6 mm) was used for stability studies in biological media, and a 5-μm Luna C18(2) column (50 × 4.6 mm) was used for solubility and chemical stability analysis. Both columns were purchased from Phenomenex. The injection volume was 20 μl, the mobile phase flow rate was 2 ml/min, and the following program was applied for the elution: 0–0.2 min, 0% B; 0.2–2.7 min, 0–100% B; 2.7–3.2 min, 100% B; 3.2–3.4 min, 100–0% B; 3.4–6.1 min, 0% B. Solvent B was HPLC grade acetonitrile (Sigma-Aldrich CHROMASOLV). The aqueous solvent contained 0.1% trifluoroacetic acid, and the detection wavelength was 365 nm.

##### Solubility

Thermodynamic solubility was measured by dissolving the compounds up to saturation in a pH 7.4 phosphate-buffered saline (PBS) with the following composition: 137.5 mm NaCl, 2.7 mm KCl, 4.3 mm Na_2_HPO_4_, and 1.4 mm KH_2_PO_4_ supplemented or not with 10% hydroxypropyl-β-cyclodextrin (HP-βCD). Samples were shaken for 24 h at 21 ± 1 °C. Saturation was confirmed by the presence of undissolved powder. After ultracentrifugation, the concentration in the supernatant was measured by an HPLC procedure using a calibration curve established for each compound by diluting a 10 mm DMSO stock solution to adapted concentrations. Due to rapid degradation of CN-chalcone 4, solubility was determined after 2 h of shaking. The indicated value given for CN-chalcone 4 is thus an estimate of the solubility.

##### Chemical Stability

Stability of compounds was assessed in PBS, pH 7.4, with or without 10% HP-βCD at 20 °C up to 24 h. For each compound, the 10 mm DMSO stock solution was diluted to a final incubation concentration of 10 μm with 0.1% DMSO. 20 μl of sample were removed at *t*_0_, 1, 2, 4, 6, 8, 10, and 24 h and directly injected onto the HPLC. The percentage of remaining test compound relative to *t*_0_ was measured by monitoring the peak area on the chromatogram.

##### Stability in Mouse Serum

Stability of chalcones was determined in mouse serum with or without 10% HP-βCD at 37 °C up to 16 h. For each compound, the 10 mm DMSO stock solution was diluted in serum to a final concentration of 20 μm with 1% DMSO. For the measurements with HP-β-cyclodextrin, a solution of PBS (pH 7.4) containing 10% (v/w) HP-βCD was saturated with compound powder. The saturated solution was then diluted in murine serum to a final compound concentration of 20 μm. The mixture was divided into five aliquots. The incubation of each aliquot was stopped at *t*_0_ and 30 min, 1, 2, and 16 h for chalcone 4 and at *t*_0_ and 15, 30, 45, and 60 min for CN-chalcone 4 by adding one volume of ice cold acetonitrile. Samples were stirred for 3 min, sonicated for 3 min, and then centrifuged at 4 °C before HPLC injection. The percentage of remaining test compound relative to *t*_0_ was measured by monitoring the peak area on the chromatogram.

##### Stability in Lung Homogenate

Stability of chalcones was determined in mouse lung homogenate with or without HP-βCD 10% at 37 °C. Lung homogenate was prepared separately with a Fastprep® (Q-BIOgene, Illkirch, France) in PBS (one lung homogenized in 1 ml of buffer). For each compound, the incubation solutions, the sampling, and the extraction conditions were prepared as described under “Stability in Mouse Serum.”

##### Cell Culture

Human embryonic kidney 293 cells expressing the fusion receptor EGFP-hCXCR4 (stable cell lines (26)) were cultured to ∼80% confluence in 75-cm^2^ flasks in minimum Eagle's medium with Earle's salt supplemented with 10% fetal calf serum, 2 mm glutamine, and 1% antibiotics (penicillin/streptomycin) and replated twice a week. HepG2 cells were grown to ∼80% confluence in 75-cm^2^ flasks in MEM with Earle's salt supplemented with 10% fetal calf serum, 2 mm glutamine, 1% antibiotics (penicillin/streptomycin), 1 mm sodium pyruvate (Invitrogen), 0.1 mm non-essential amino acids (Invitrogen) and replated twice a week.

##### Binding Experiments

FRET-based binding experiments were carried out as described (26, 46). Human embryonic kidney 293 cells expressing the fusion receptor EGFP-hCXCR4 were harvested in PBS (137 mm NaCl, 2.7 mm KCl, 4.3 mm Na_2_PO4·7H2O, 1.4 mm KH_2_PO_4_, pH 7.4) supplemented with 5 mm EDTA, pH 7.4, centrifuged, and resuspended in HEPES-bovine serum albumin (BSA) buffer (10 mm HEPES, 137.5 mm NaCl, 1.25 mm MgCl_2_, 1.25 mm CaCl_2_, 6 mm KCl, 10 mm glucose, 0.4 mm NaH_2_PO_4_, 0.1% bovine serum albumin (w/v), pH 7.4). Cells were used at a concentration of 10^6^ cells/ml. Apparent affinities of the different ligands were determined by real-time fluorescence monitoring ligand-receptor interactions. Time-based recording of binding was initiated by adding 100 nm CXCL12-TR (Texas Red-labeled CXCL12) to the 0.5-ml cell suspension. Fluorescence emitted at 510 nm (excitation at 470 nm) was recorded at 21 °C using a Fluorolog 2 spectrofluorimeter (SPEX) and sampled every 0.3 s. Binding of CXCL12-TR to EGFP-labeled CXCR4 was detected as a reversible decline of emission at 510 nm, due to energy transfer from excited EGFP to TR. For competition experiments, the fluorescent chemokine was preincubated for 1 h at room temperature with or without various concentrations of each chalcone. Then the premix was added, and fluorescence was recorded until equilibrium was reached (300 s). Data were analyzed using Kaleidagraph 3.08 software (Synergy Software, Reading, PA).

##### cAMP Determination

CXCR4 receptor coupling to adenylyl cyclase was assessed by measuring the dose-dependent inhibitory effects of chemokine with or without chalcone on forskolin-stimulated cAMP accumulation. To facilitate studies of cAMP regulation, we used a developed bioluminescence resonance energy transfer (BRET) sensor for cAMP, CAMYEL (cAMP sensor using YFP-Epac-RLuc), which can quantitatively and rapidly monitor intracellular concentrations of cAMP in cells (47). HEK EGFP-hCXCR4 cells were cultured to ∼80% confluence in 75-cm^2^ flasks in MEM with Earle's salt supplemented with 10% fetal calf serum, 2 mm glutamine, and 1% antibiotics (penicillin/streptomycin). Cells were plated on 10-cm plates and transfected with 10 μg of CAMYEL plasmid using the calcium phosphate precipitation method. Two days later, cells were plated in 96-well solid white tissue culture plates (Greiner) at a density of 60,000 cells/well the day before cAMP assay. Cells were serum-starved in Hanks' balanced salt solution, pH 7.4 (Sigma-Aldrich) for 30 min at 37 °C before treatments (80 μl/well). The BRET assay was carried out with a Victor Light plate reader (PerkinElmer Life Sciences). Emission signals from *Renilla* luciferase and YFP were measured sequentially using a BRET1 filter set (475-30/535-30). Stimulations were initiated by injection of 20 μl of 5× concentrated ligands prepared in Hanks' balanced salt solution supplemented with 500 mm isobutylmethylxanthine. After 5 min, 10 μl of a Hanks' balanced salt solution of coelenterazine-H (2 μm) were added, and plates were incubated for 10 min at 37 °C before reading.

##### Cytotoxicity

The capacity of the cells to carry out reduction reactions after drug treatment was estimated with the fluorescent Alamar Blue reagent (AbD Serotec, Oxford, UK). Proliferating cells cause the change from the oxidized blue and non-fluorescent Alamar Blue form (resazurin) to a reduced, pink, highly fluorescent form (resorufin) that can be detected using fluorescence monitoring (excitation 560 nm; emission 590 nm). HepG2 or HEK 293 cells were seeded at a density of 30,000 cells/well on a 96-well plate and allowed to settle overnight before treatment. The dye was added to the cells together with the test substances (5-fold concentrated stock solution prepared in culture medium or in PBS with 10% HP-βCD), and the fluorescence was measured after 24-h incubation, using a Flex Station (Molecular Devices).

##### Murine Model of Hypereosinophilia

The *in vivo* activity of the compounds was assessed in an 8-day model of hypereosinophilia created by immunization and challenge to ovalbumin in mice. Briefly, 9-week-old male BALB/c mice were sensitized by intraperitoneal injection of a mixture of 50 μg of ovalbumin (OVA, grade V; Sigma-Aldrich) adsorbed on aluminum hydroxide (2 mg; Sigma-Aldrich) in 0.1 ml of saline on days 0, 1, and 2. Mice were challenged intranasally with 10 μg of OVA in 25 μl of saline (12.5 μl/nostril) on days 5, 6, and 7. Control mice received intraperitoneal and intranasal administrations of saline alone. Intranasal administrations were performed under anesthesia with 50 mg/kg ketamine and 3.33 mg/kg xylazine given intraperitoneally. Food and water were supplied *ad libitum*. Animal experimentation was conducted with the approval of the government body that regulates animal research in France. The chalcone compounds were administered either by the intraperitoneal or intranasal routes. In a first set of experiments, mice received either chalcone 4 or carbonitrile-chalcone 4 solubilized in PBS with 10% HP-βCD (C0926, Sigma) by intranasal injection 2 h before each nasal OVA or saline challenge. In a second set of experiments, mice received CN-chalcone 4, vanillin, or parachlorobenzoylacetonitrile intranasally, in PBS with HP-βCD 10%, 2 h before each OVA or saline challenge. In the last set of experiments, mice received chalcone 4 or CN-chalcone 4 in suspension in carboxymethylcellulose 1% or solubilized in PBS with HP-βCD 10% and administered by intraperitoneal injection 2 h before each OVA or saline challenge. Collection of bronchoalveolar lavage fluid (BALF) was performed 24 h after the last OVA challenge. Mice were deeply anesthetized by intraperitoneal injection of 150 mg/kg ketamine and 10 mg/kg xylazine. A plastic canula was inserted into the trachea, and airways were lavaged by 10 instillations of 0.5 ml of ice-cold saline supplemented with 2.6 mm EDTA (saline-EDTA). Total and differential cell counts in the BALF were performed after centrifugation (300 × *g* for 5 min at 4 °C) to pellet cells. Erythrocytes were lysed by hypotonic shock by the addition of 1.5 ml of distilled H_2_O, followed by the addition of 0.5 ml of 0.6 m KCl. Cells were centrifuged and resuspended in 500 μl of ice-cold saline-EDTA, and total cell counts were determined using a hemocytometer (Neubauer's chamber). Differential cell counts were assessed on cytologic preparation obtained by cytocentrifugation (Cytospin 3, Shandon Ltd.) of 200 μl of diluted BALF (250,000 cells/ml in ice-cold saline-EDTA). Slides were stained with Hemacolor (Merck), and counts were performed on at least 400 cells for each preparation. Differential counts were expressed as absolute numbers or as a percentage of the total number of cells.

##### Cytochrome c Oxidase Activity

Lung and heart were homogenized separately with an UltraTurax® (IMLAB, Lille, France) in PBS, pH 7.4 (one organ in 1 ml of buffer). Lung homogenate was diluted at 1:2 and heart homogenate at 1:5 in PBS. 100 μl of each diluted homogenate were placed in a microplate, and 100 μl of assay reagent (10 mm tetramethyl-*p*-phenylenediamine, 2 mm sodium ascorbate, 20 mm KH_2_PO_4_) were added. The microplate was placed immediately in a microplate spectrophotometer reader, and optical density kinetic (λ = 610 nm) was measured every 20 s for 30 min. Results are expressed as *V*_max_/100 μg of protein (OD/min/100 μg of protein).

## RESULTS

### 

#### 

##### Carbonitrile-chalcone 4 Neutralizes CXCL12 in Vitro and in Vivo

**FIGURE 1.**
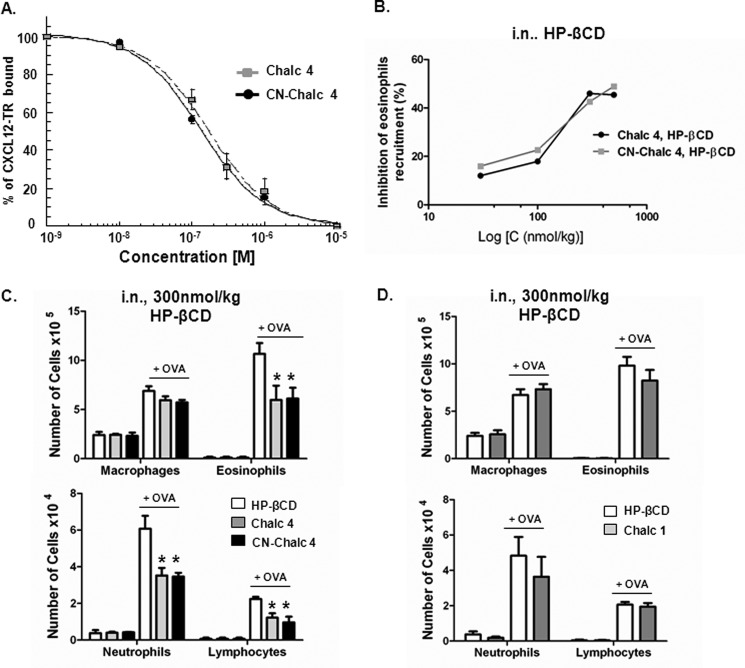
**Carbonitrile-chalcone reduces inflammation in a mouse model of allergic eosinophilic airway inflammation.** *A*, *in vitro* inhibition of CXCL12 binding to CXCR4 receptor by chalcone 4 (*Chalc 4*) and CN-chalcone 4 (*CN-Chalc 4*). Inhibition of CXCL12 binding to CXCR4 as a function of increasing concentration of chalcone 4 (*gray squares*) and CN-chalcone 4 (*black circles*) is monitored using FRET intensity variation. The fluorescence of cells expressing EGFP-labeled CXCR4 is followed at 510 nm as a function of time. Upon the addition of Texas Red-labeled CXCL12 (*CXCL12-TR*, 100 nm), fluorescence intensity at 510 nm declines as a result of interaction between CXCL12-TR and EGFP-CXCR4, which causes FRET. The *ordinate axis* reports the intensity of FRET as a percentage of the control value (100 nm CXCL12-TR alone). *K_i_* values were derived from the IC_50_ values determined from competition curves using the Cheng and Prusoff relationship (65). *K_i_* values are 53 ± 31 nm (chalcone 4) and 45 ± 57 nm (CN-chalcone 4). Each data point represents the mean ± S.D. (*error bars*) of three experiments. *B*, *in vivo* dose-response effect of a topical treatment with chalcone 4 and CN-chalcone 4 in an 8-day mouse model of hypereosinophilia. BALB/c mice were sensitized and challenged with OVA or saline. Chalcone 4 (*gray line*) or CN-chalcone 4 (*black line*) solubilized in 10% HP-βCD were administered intranasally 2 h before each challenge. The percentage of inhibition of eosinophil is shown. Data points (*squares*) are means of *n* = 6 determinations. *C* and *D*, effect of topical (intranasal; *i.n.*) treatment with chalcone 4, CN-chalcone 4 (*C*), and chalcone 1 (*Chalc 1*) (*D*) in the 8-day mouse model of hypereosinophilia. BALB/c mice were sensitized and challenged with OVA or saline. Drugs (300 nmol/kg) were administered intranasally 2 h before each challenge in HP-βCD 10% (vehicle). Absolute numbers of macrophages, eosinophils, neutrophils, and lymphocytes in BALF are shown. *Bars* represent means, and *error bars* show S.E. values (*n* = 6/group). *, *p* ≤ 0.05 in comparison with the saline-treated OVA group.

CN-chalcone 4 was prepared by condensing *para*-chlorobenzoyl-acetonitrile (pCBA) with protected vanillin (*VanP*) as described in Scheme 1. CN-chalcone 4 is the homolog of chalcone 4, formerly described as a ligand of CXCL12, that inhibits chemokine binding to both CXCR4 and CXCR7 receptors (21, 23, 26). CN-chalcone 4 prevents binding of Texas Red-labeled CXCL12 (CXCL12-TR, 100 nm) to EGFP-tagged CXCR4, determined by fluorescence resonance energy transfer (26) (Fig. 1*A*) with similar affinity as chalcone 4 (*K_i_* = 45 ± 57 nm for chalcone 4 *versus K_i_* = 53 ± 31 nm for CN-chalcone 4). This inhibition of CXCL12 binding to CXCR4 consequently blocks CXCL12-evoked CXCR4 cellular signaling (inhibition of cAMP production, shown in Fig. 4*B*) and trafficking (data not shown).

The *in vivo* activity of CN-chalcone 4 was assayed in a recently developed 8-day mouse model of airway hypereosinophilia (24). In this model, mice are sensitized by intraperitoneal injection of OVA (50 μg) adsorbed on 2 mg of aluminum hydroxide in 0.1 ml of saline on days 0, 1, and 2. Mice are then challenged intranasally with 10 μg of OVA in 25 μl of saline (12.5 μl/nostril) on days 5, 6, and 7. Drugs to be tested can be administered either systemically by the intraperitoneal route or locally by the intranasal route. The intranasal route mimics the inhalation exposure used in humans, which is the preferred and well accepted administration for inflamed airway treatment, in particular asthma. Thus, unless otherwise stated, drugs were administered intranasally in this study. Due to limited solubility of chalcone 4 (9 ± 1 μm) and CN-chalcone 4 (16 ± 2 μm) in saline buffer, drugs were dissolved in physiological solutions complemented with HP-βCD (10%, w/w). Under such conditions, chalcone 4 and CN-chalcone 4 could be dissolved at maximal concentrations reaching 690 ± 44 and 493 ± 36 μm, respectively.

Administering these solutions at 25 μl/mouse intranasally allows a maximal dose approximating 300–500 nmol/kg. Eosinophil counts in BALF are dose-dependently inhibited by both chalcone 4 and CN-chalcone 4 up to 50% at doses of 300–500 nmol/kg, which can be reached as limits of drug solubility (Fig. 1*B*).

BALF infiltrate with macrophages, eosinophils, neutrophils, and lymphocytes was determined under treatment with intranasal chalcone 4 and CN-chalcone 4 at 300 nmol/kg. Fig. 1*C* shows that neither vehicle (10% HP-βCD) nor any of the chalcones by themselves elicit any cell recruitment in the airways. After ovalbumin challenge, a significant increase in the number of eosinophils and macrophages occurs (Fig. 1*C*, *top*). Polymorphonuclear neutrophils and lymphocytes were also significantly present in BALFs after OVA challenge, albeit at ∼10–20-fold lower levels than eosinophils (Fig. 1*C*, *bottom*). Significant reduction of eosinophil recruitment is noted with chalcone 4 administered intranasally (300 nmol/kg). In addition, we note that the new molecule CN-chalcone 4 is as potent as chalcone 4.

In order to further document the specificity of chalcone 4 and CN-chalcone 4 action, we tested the activity of the unsubstituted chalcone backbone (*chalc 1* in Scheme 1) already reported as inactive upon CXCL12 binding to CXCR4 (26). Fig. 1*D* shows that chalcone 1 does not promote any inflammatory response *per se*; nor does it significantly attenuate eosinophil or any other inflammatory cell recruitment in the airways.

These results therefore indicate that the inactive chalcone chemotype can be substituted by functional groups to inhibit CXCL12 binding to CXCR4 to become active as an attenuator of allergen-induced inflammatory responses. CN-chalcone 4 is as active as chalcone 4 to neutralize CXCL12 *in vitro* and to inhibit eosinophilic airway inflammation *in vivo*. This shows that the introduction of the carbonitrile group in chalcone 4 does not affect the activity and potency of the compound.

##### CN-chalcone 4 Is Active Locally but Not Systemically

**FIGURE 2.**
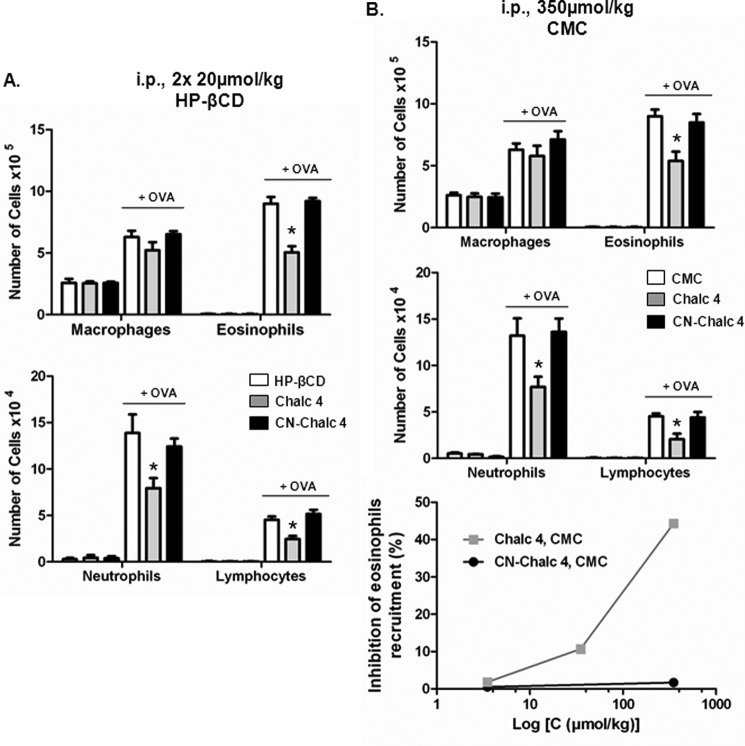
**CN-chalcone 4 is an antedrug.** Systemic effect of chalcone 4 and CN-chalcone 4 in two vehicles. Systemic (intraperitoneal (*i.p.*)) treatment with chalcone 4 (*Chalc 4*) and CN-chalcone 4 (*CN-Chalc 4*) in the 8-day mouse model of hypereosinophilia is shown. BALB/c mice were sensitized and challenged with OVA or saline. Drugs (two administrations of 20 μmol/kg/day for 3 days in 10% HP-βCD (vehicle) (*A*) or 350 μmol/kg once a day for 3 days in 1% carboxymethylcellulose (*CMC*) as a vehicle (*B*)) were administered intraperitoneally 2 h before each OVA challenge. Absolute numbers of macrophages, eosinophils (*top*), neutrophils, and lymphocytes (*middle*) in BALF are shown. *Bars* show means, and *error bars* show S.E. values (*n* = 6/group). *, *p* ≤ 0.05 in comparison with the saline-treated OVA group. Dose intensity relationship of eosinophil recruitment in the intraperitoneal route is shown for chalcone 4 and CN-chalcone 4 up to the maximal dose (*bottom*).

We then compared the *in vivo* activity of CN-chalcone 4 administered systemically by the intraperitoneal route with that of chalcone 4 (Fig. 2). Chalcone 4 and CN-chalcone 4 were solubilized in HP-βCD (10%), or in carboxymethylcellulose, allowing administrations either of 2 × 20 μmol/kg (Fig. 2*A*) or of a 350 μmol/kg (Fig. 2*B*) dose of compound, respectively. Chalcone 4 and CN-chalcone 4 could thus be administered intraperitoneally at doses 40- or 700-fold higher than those used for intranasal administration. These doses correspond to the maximal possible dose because the solubility of chalcones is limited by the concentration of vehicle (carboxymethylcellulose or HP-βCD) and because higher doses of vehicle lead to toxic effects on animals. Fig. 2 displays results of inflammatory cell recruitment in BALF of OVA-sensitized mice receiving the compounds or vehicles.

Fig. 2*A* shows the effect of twice daily treatment with 20 μmol/kg chalcone 4 or CN-chalcone 4 in 10% HP-βCD administered intraperitoneally. Neither HP-βCD alone nor any of the two drugs had any effect on inflammatory cell recruitment (macrophages, eosinophils, neutrophils, or lymphocytes) in the naive airways. When administered 1 h before the challenging doses of OVA, chalcone 4 shows anti-inflammatory properties by significantly reducing eosinophils, neutrophils, and T cell counts in BALF (Fig. 2*A*). By contrast, CN-chalcone 4 did not affect any inflammatory cell counts. Repeating the experiment with carboxymethylcellulose as the excipient allowed administration at doses as high as 350 μmol/kg (Fig. 2*B*). Again, only chalcone 4 exhibits dose-dependent anti-inflammatory activity in the airways with significant inhibition of macrophage recruitment in addition to inhibition of eosinophil, neutrophil, and lymphocyte influx. These experiments reveal that, in contrast to chalcone 4, CN-chalcone 4 is inactive at inhibiting airway inflammation *in vivo* when administered at a distance from the airways. Therefore, introducing a carbonitrile group in chalcone 4 affects its distribution or metabolism.

##### CN-chalcone 4 Is Rapidly Degraded in Biological Media

**FIGURE 3.**
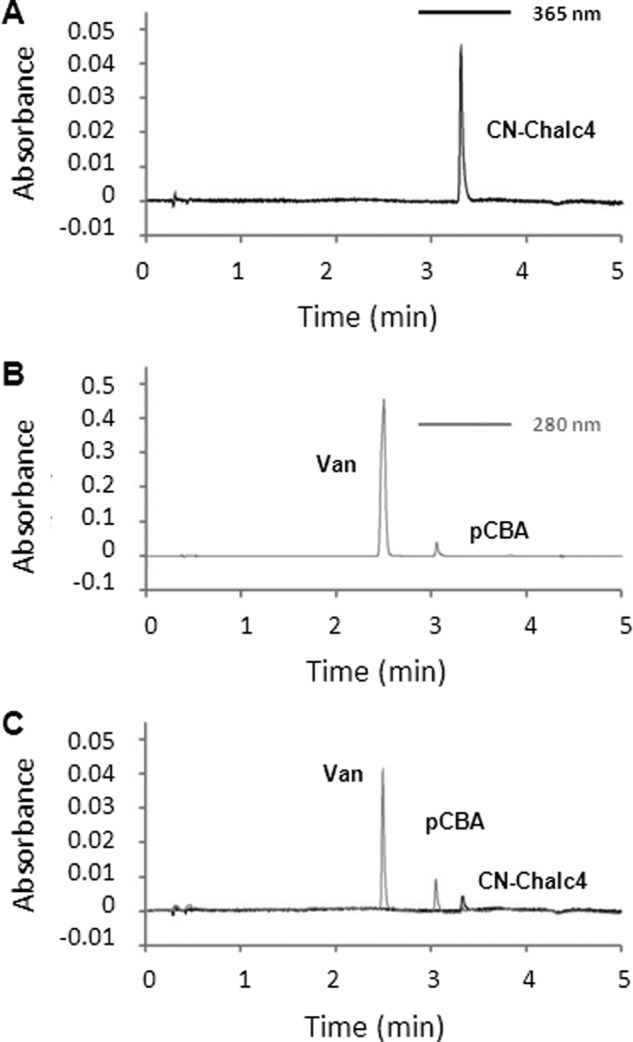
**CN-chalcone 4 is rapidly hydrolyzed in murine serum.** Chromatograms were obtained with a Luna C18(2) 5-μm, 4.6 × 50-mm column. Gradient elution was as follows: 0–0.2 min, 0% B; 0.2–2.7 min, 0–100% B; 2.7–3.2 min, 100% B; 3.2–3.4 min, 100–0% B; 3.4–6.1 min, 0% B (A, water, 0.1% trifluoroacetic acid; B, acetonitrile). *A*, chromatogram (at λ = 365 nm) of 20 μl of the reference compound CN-chalcone 4, dissolved at 10 μm in water/acetonitrile (1:1, v/v) (*t_R_* = 3.31 min). *B*, chromatogram (at λ = 280 nm) of 20 μl of the reference compounds vanillin (*Van*) and pCBA, dissolved at 100 μm in water/acetonitrile (1:1, v/v) (*t_R_*(Van) = 2.47 min and *t_R_*(pCBA) = 3.02 min). *C*, chromatogram (at λ = 280 nm and 365 nm) of 20 μl of CN-chalcone 4 dissolved at 20 μm and incubated for 1 h in murine serum. The solution was diluted with one volume of acetonitrile before injection in the HPLC. Peaks detected at *t_R_* = 2.47 min, *t_R_* = 3.02 min, and *t_R_* = 3.31 min correspond to vanillin, pCBA, and CN-chalcone 4 (*CN-Chalc4*), respectively.

One major effect of the introduction of the carbonitrile group on chalcone 4 is detected on the stability of CN-chalcone 4 in biological media. Compound stability was assessed using HPLC detection (Fig. 3) after incubation in various media. In contrast to chalcone 4, which is stable for hours in buffer and tissue homogenates (Table 1), CN-chalcone 4 is rapidly degraded in phosphate-buffered saline (*t*½ = 6 h) and even more rapidly in murine serum (*t*½ = 20 min) or lung homogenate (*t*½ = 25 min). 10% HP-βCD not only solubilizes the molecule but also significantly improves its stability. CN-chalcone 4 half-life reaches 40 min in lung homogenate in the presence of HP-βCD. Whatever the experimental condition, the half-life of CN-chalcone 4 is significantly shorter than that of chalcone 4, possibly accounting for the lack of effect when using the intraperitoneal route rather than the intranasal administration.

**TABLE 1:** **Stability of chalcone 4 *versus* CN-chalcone 4 in different media** Compound stability was measured either in HEPES buffer, pH 7.4, or murine serum, or lung homogenates supplemented or not with 10% HP-βCD, as indicated. Incubation of compound was stopped at 15 and 30 min and 1, 2, 16, and 24 h, depending on the compound. Values in parentheses represent the percentage of starting molecule recovered at the indicated time.

	Stability half-life (*t1/2*)	
	Chalcone 4	CN-chalcone 4
	*h*	
PBS	>10 (98% at 10 h)*^a^*	6
PBS, 10% β-cyclodextrine	>10 (95% at 10 h)*^a^*	16.3
Murine serum	>16 (96% at 16 h)	0.3
Murine serum, 10% β-cyclodextrine	>16 (91% at 16 h)	1.3
Lung homogenate	>6 (60%)*^a^*	0.4
Lung homogenate, 10% β-cyclodextrine	>16 (66% at 16 h)	0.6

*^a^* Data are taken from Ref. 24.

**SCHEME 2.**
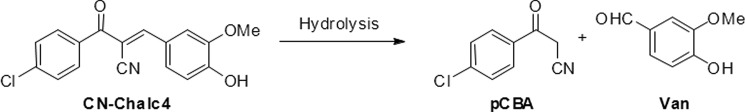
**Hydrolysis reaction of CN-chalcone 4 toward pCBA and vanillin (*Van*).**

Because the carbonitrile group is an electron-attracting group, its presence on CN-chalcone 4 facilitates nucleophilic attack by water molecules, a reaction that ultimately leads to the hydrolysis of the molecule and production of pCBA and vanillin (*Van*) as shown in Scheme 2. This is indeed observed on reverse-phase HPLC chromatograms (Fig. 3*B*), showing that hydrolysis of CN-chalcone 4 is accompanied by the concomitant appearance of its two constituents, which represent the major degradation products in biological media (Fig. 3*C*).

**FIGURE 4.**
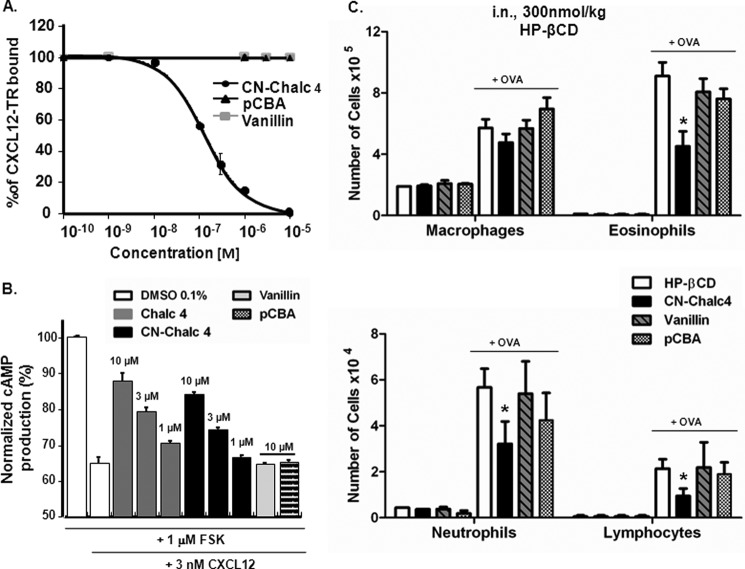
**CN-chalcone 4 degradation products are inactive.** *A*, CN-chalcone 4 (*CN-Chalc 4*) degradation products (up to 10 μm) do not inhibit CXCL12-TR binding to EGFP-CXCR4 detected by FRET as in Fig. 1*A*. Each data point represents the mean ± S.D. of three independent experiments performed in triplicates. *B*, CN-chalcone 4, but not its degradation products, dose-dependently inhibits CXCL12 action on forskolin-evoked cAMP responses in HEK EGFP-CXCR4 cells. The *first two bars* report the maximal production of cAMP (%) triggered by 1 μm forskolin and its inhibition by 3 nm CXCL12. The *following bars* show that chalcone 4 (*Chalc 4*) and CN-chalcone 4 (1, 3, and 10 μm), but not vanillin or pCBA (10 μm), inhibit the CXCL12 effect on cAMP production. Each *bar* represents the mean ± S.D. (*error bars*) of three independent experiments performed in triplicates. *C*, topical treatment with chalcone 4, CN-chalcone 4, vanillin, and pCBA in the 8-day mouse model of hypereosinophilia. BALB/c mice were immunized and challenged with OVA or saline. Treatments (300 nmol/kg) or HP-βCD 10% (vehicle), were administered intranasally 2 h before each challenge. Absolute numbers of macrophages, eosinophils, neutrophils, and lymphocytes in BAL are shown. *Bars* show means, and *error bars* show S.E. values (*n* = 6/group). *, *p* ≤ 0.05 in comparison with the saline-treated OVA group.

Because CN-chalcone 4 degradation occurs rapidly, we investigated the biological activity of its degradation products with regard to CXCL12 neutralization, cell signaling, and *in vivo* inhibition of eosinophil recruitment in the airways. Fig. 4*A* shows that CN-chalcone 4 dose-dependently prevents CXCL12 binding to CXCR4 with maximal inhibition beyond 1 μm. Neither vanillin nor pCBA exhibits any binding-neutralizing activity at concentrations up to 10 μm, indicating that binding inhibition is indeed due to CN-chalcone 4 itself.

Inhibition of CXCL12 effect on cAMP formation in transfected HEK293 cells overexpressing the human CXCR4 receptor has also been characterized. As shown in Fig. 4*B*, forskolin (1 μm) evokes an increase in intracellular cAMP that is potently blocked by CXCL12 (3 nm). This blocking effect of CXCL12 is dose-dependently counteracted by either chalcone 4 or CN-chalcone 4 (1–10 μm) but not by the CN-chalcone 4 degradation products vanillin (10 μm) and pCBA (10 μm). The derived IC_50_ values are equal to 4.1 ± 0.3 and 6.9 ± 0.4 μm for chalcone 4 and CN-chalcone 4, respectively.

*In vivo* effects of the degradation products have also been tested on OVA-sensitized and -challenged mice. Intranasal administration of vanillin and pCBA at the same dose as CN-chalcone 4 in Fig. 1 (300 nmol/kg) has no effect on eosinophil recruitment in the airways (Fig. 4*C*), showing their lack of activity *in vivo* that matches their lack of *in vitro* activity on CXCL12.

Thus, CN-chalcone 4, which is as active as chalcone 4, is subject to spontaneous hydrolysis in buffered aqueous solutions and biological fluids. Its hydrolysis products, vanillin and pCBA, show no binding activity toward CXCL12 or CXCR4 and no biological activity either on cells or in the inflamed airways in our mouse model. All observed effects are therefore due to CN-chalcone 4 itself before degradation occurs. The short half-life of CN-chalcone 4 suggests that it may not diffuse over a long distance *in vivo*. This would result in the neutralization of CXCL12 only in tissues directly exposed to CN-chalcone 4, possibly those in the immediate vicinity of the administration site. This is the most plausible explanation of the *in vivo* activity observed after intranasal administration of CN-chalcone 4 and of the lack of activity of CN-chalcone 4 administered systemically by intraperitoneal injection.

##### Chalcone 4, CN-chalcone 4, and Its Hydrolysis Products Have Low Toxicity

**FIGURE 5.**
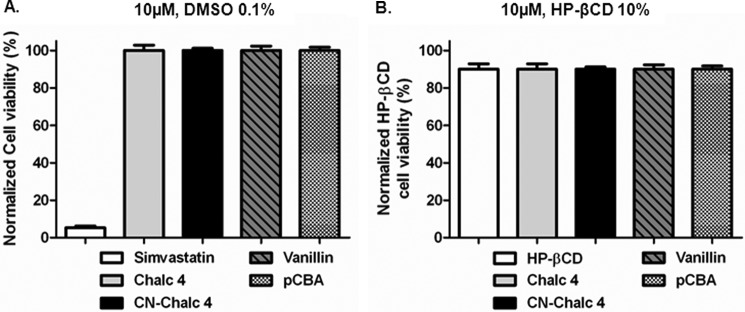
**Cytotoxicity of chalcone derivatives on HepG2 cells using a resazurin reduction assay.** Cell viability is expressed as the percentage of untreated control cells (*A*) and as 10% HP-βCD-treated cells (*B*). Cells were exposed to 10 μm compounds for 24 h. Each exposure was preceded by equilibrium setting for 16 h in growth medium at 37 °C, 5% CO_2_. The dye was added to the cells together with the test substances at a final concentration of 10%. Simvastatin (100 μm) was used as a positive control. All samples contained 0.1% DMSO. Data are expressed as means ± S.D. (*error bars*) (*n* = 3). *Chalc 4*, chalcone 4; *CN-Chalc 4*, CN-chalcone 4.

We checked for cytotoxicity of chalcone 4, CN-chalcone 4, and its hydrolysis products vanillin and pCBA by measuring mitochondrial reduction of the Alamar Blue dye (Fig. 5*A*). HepG2 cells were incubated for 24 h with chalcone 4, CN-chalcone 4, vanillin, or pCBA at a 10 μm concentration. The positive cytotoxicity control molecule was simvastatin (100 μm) (48).

In order to increase the low chalcone 4 solubility (9 ± 1 μm in physiological medium), we also used HP-βCD in this study. Although HP-βCD displays some toxicity on its own at 10% in culture medium, as was described previously (66, 67), there was no further cytotoxicity of any of the compounds (Fig. 5*B*) as compared with cells treated with HP-βCD alone.

General toxicity was evaluated *in vivo* in mice that received CN-chalcone 4 intraperitoneally at a dose of 350 μmol/kg (100 mg/kg) per day during three consecutive days. As reported in Table 2, there was no body or spleen weight loss nor any modification of cytochrome *c* oxidase activity in lung and heart, indicating no toxicity of CN-chalcone 4 administered at the highest active dose.

**TABLE 2:** **General toxicity** Absence of general toxicity of CN-chalcone 4. After OVA challenge leading to hypereosinophilia and CN-chalcone 4 treatment, whole animals and spleens were weighed. Whole animal weights were compared with those of untreated animals (body weight as a percentage of that for control animals). Spleen weight is given in mg. Cytochrome *c* oxidase activity was determined in lung and heart, and maximal activity (*V*_max_) is reported as the variation of tetramethyl-*p*-phenylenediamine 610-nm optical density/min/100 μg of protein.

	Weight		Cytochrome *c* oxidase (*V*_max_)	
	Body	Spleen	Lung (×10^2^)	Heart (×10^3^)
	%	*mg*		
Carboxymethylcellulose	99.9 ± 4	124 ± 7	15.1 ± 1.6	25.6 ± 2.6
CN-chalcone 4	99.6 ± 6	125 ± 11	13.8 ± 2.8	28.5 ± 3.2
OVA	99.8 ± 6	128 ± 6	12.8 ± 2.0	23.1 ± 3.1
OVA+ CN-chalcone 4	99.6 ± 6	123 ± 4	12.7 ± 1.6	29.9 ± 3.5

## DISCUSSION

Our results show the anti-inflammatory effect of a rapidly hydrolyzable CXCL12 neutraligand in an airway hypereosinophilia model. Carbonitrile-chalcone 4 is an efficient blocker of CXCL12 binding to CXCR4 and of the associated inhibition of cAMP production. However, in biological fluids, CN-chalcone 4 is rapidly degraded into two inactive metabolites, vanillin and pCBA, the two compounds that served as synthetic building blocks for its production. When administered locally in the airways by the intranasal route, CN-chalcone 4 efficiently inhibits eosinophil, neutrophil, and T cell recruitment at a low dose. By contrast, it remains without any anti-inflammatory effect in the airways when administered systemically by the intraperitoneal route even at doses 100–1000-fold higher. This is opposed to the systemic effect of chalcone 4 and demonstrates that CN-chalcone 4 behaves as an antedrug or soft drug acting at the administration site that is degraded prior to wider distribution.

Three groups, including ours (24–27), described that when CXCR4 signaling is inhibited, either with antibodies (25), with CXCR4 antagonists (27), or with CXCL12-neutralizing small molecules (26), invasion of lungs by eosinophils is reduced by ∼50%. This piece of evidence highlights a functional role of CXCR4 and of its ligand either in the allergic response onset or in its maintenance. The question as to whether airway inflammation stimulates CXCL12 production continues to be debated because immunohistochemical detection in lung tissue shows no change (25), whereas immunochemical determination in BALF (49) and gene expression in lung (50) indicate that CXCL12 is up-regulated. The expression of CXCR4, on the other hand, is higher in BAL CD4^+^ T cells of human asthmatics as compared with their peripheral blood CD4^+^ lymphocytes (51) and is up-regulated by the proinflammatory cytokine IL-4 in CD4^+^ T cells, including Th2 cells (25, 52–54). This renders significant response to CXCL12 likely to occur in the airway, whatever the regulation of CXCL12 expression. In addition, CXCR4 is also expressed in eosinophils (55, 56). Eosinophils have a migratory response to CXCL12 comparable with that evoked by eotaxin.

The mode of action of neutraligands opens the way to new therapeutic strategies especially for airway diseases, because (i) chalcone 4 and its analogs are active through the intranasal route, and (ii) they act on a new target, namely CXCL12, the ligand of CXCR4 and CXCR7 chemokine receptors. Thus, the mode of action of chalcone 4 (26) and its analogs chalcone 4-phosphate (24) and CN-chalcone 4 (this work) appears as complementary to that of classical receptor antagonists because the blockade of the chemokine is without any effect on the receptor. In particular, it is neither a partial agonist of CXCR4 nor an activator of CXCR7 (42, 57, 58), as was described for AMD 3100 and in other instances with RANTES (regulated on activation normal T cell expressed and secreted) analogs acting on the CCR5 receptor (59). Therefore, the mechanism of action of chalcone 4 and its analogs deserves to be exploited in drug development programs.

Another concern was raised regarding the large tissue distribution of CXCR4, which can be the cause of possible side effects of CXCR4-targeting drugs. The use of systemically administered AMD 3100 confirmed the risk of side effects resulting from general CXCR4 inhibition. This was illustrated on leukocyte maturation in the bone marrow (27) and on cardiac function (36, 37, 60). We therefore generated a short lived readily hydrolyzable analog of the initial compound, chalcone 4, and show here that CN-chalcone 4 is as active as chalcone 4 on airway inflammation when administered by the intranasal route, whereas it is inactive when delivered systemically using the intraperitoneal route. It therefore typically behaves as an antedrug or soft drug.

The general principles and reactions that are used for antedrug structures include various cleavable chemical functions, such as carboxylic esters and amides, oximes, thioester, spiroenones, or lactones (45, 61). In designing carbonitrile-chalcone 4, we here make use of the Knoevenagel and retro-Knovenagel reactions (62) yielding the desired compound due to a reversible aldolization reaction (63) that has never been exploited in the antedrug field before. The biologically active compound, carbonitrile-chalcone 4, is readily hydrolyzed in aqueous media with a half-life of a few tens of min and yields vanillin and pCBA, which both serve as synthetic building blocks for the preparation of carbonitrile-chalcone 4. The probable hydrolysis mechanism involves the addition of one water molecule according to a Michael addition on the α-β unsaturated conjugated system. Hydration of the double bond is presumably facilitated by the presence of the electron-attracting nitrile group. The resulting enolic structure then evolves toward production of the initial reactants vanillin and pCBA according to a retroaldolization reaction (64). We show here that neither the reactants nor carbonitrile-chalcone 4 display any toxic effect *in vivo* or in HepG2 cells *in vitro*.

In conclusion, our results show a strong activity of a chalcone 4 derivative, carbonitrile-chalcone 4, displaying only local and no systemic effect due to a short lifetime in biological fluids, therefore playing the role of an antedrug, which is particularly interesting when the airways are considered. The various chalcone 4 derivatives that we have generated in this and previous works will serve as tools to understand CXCR4, CXCR7, and CXCL12 functions in the airway inflammation process. In particular, the sequence of events and their dependence on CXCL12 activity will be important elements in the characterization of CXCL12 as a drug target in airway inflammation. The mechanism of action of chalcone 4 and its analogs deserves to be exploited in drug development programs because blockade of the chemokine is without any effect on the receptor spontaneous activity as opposed to the most widely encountered pharmacological action of G protein-coupled receptor antagonists.

## Footnotes

6 The abbreviations used are:

CN-chalcone 4: carbonitrile-chalcone 4
BALF: bronchoalveolar lavage fluid
BRET: bioluminescence resonance energy transfer
HP-βCD: hydroxypropyl-β-cyclodextrin
OVA: ovalbumin
TR: Texas Red
pCBA: *para*-chlorobenzoylacetonitrile.
